# Consistency of Delivery Mode Increases Chinese Mothers’ Maternal Satisfaction: The Effect of Perception of Support from Medical Staff and Mothers’ Self-Efficacy

**DOI:** 10.3390/ijerph192214904

**Published:** 2022-11-12

**Authors:** Nan Zhang, Shanshan An

**Affiliations:** 1 School of Economics and Management, Beijing Jiaotong University, Beijing 100044, China; 2 School of Law, Jiangnan University, Wuxi 214122, China

**Keywords:** maternal satisfaction, consistency of delivery mode, perception support from medical staff, self-efficacy, ethics in childbirth

## Abstract

Maternal satisfaction is essential for women. Extant research has focused on how a practical delivery method effects maternal satisfaction. This article tried to explore the effect of the consistency of delivery mode between mothers’ expectations and their experience of maternal satisfaction and proposed the mediating effect of the perception of support from medical staff and the moderated mediation effect of maternal self-efficacy. Based on two studies, this article found that the consistency of the delivery mode has a positive effect on maternal satisfaction, and women’s perception of support from medical staff mediated the above relationship. The maternal perception of self-efficacy has a moderated mediation effect; specifically, for women with a high level of self-efficacy, the positive effect of the consistency of the delivery mode on maternal satisfaction through perception support from medical staff is stronger. This article highlights the importance of the consistency of the delivery mode between women’s expectations and the experience of maternal satisfaction and the psychological mechanisms involved. The results extend the theoretical research on ethics in childbirth and provide implications for improving women’s maternal satisfaction from medical staff and themselves.

## 1. Introduction

Maternal satisfaction is the most direct and important evaluative indicator for the childbirth process and healthcare services. It refers to the positive evaluation of different dimensions of the childbirth experience [1]. A number of studies have shown that maternal satisfaction could affect the mother’s physical and mental health, the relationship with their baby, and the willingness to give birth again [2,3,4,5]. Women with high childbirth satisfaction have a good sense of self and can adapt to the role of mother more quickly [2]. In contrast, women with low childbirth satisfaction are prone to postpartum depression, child abuse, marital discord, violent injuries, and even medical litigation [6,7,8].

Women’s maternal satisfaction has been proven to be influenced by multiple variables. On the one hand, it comes from the relevant objective conditions of the maternal delivery process, including pain in labor, self-rated physical health, delivery method, medical intervention, and mother and child safety [9,10,11]. On the other hand, it is subject to many subjective cognitive and emotional factors during the labor process, such as the mother’s expectations, fear of pain, childbirth self-efficacy, participation in decision making, and maternal perception support from medical staff [1,4,11,12,13,14]. 

Extant literature has primarily focused on the actual delivery method of the parturient (cesarean section or vaginal delivery) and its impact on childbirth satisfaction [15,16]. However, little research has paid attention to the consistency between the expected and actual delivery method and its effect on women’s childbirth satisfaction. Inconsistent delivery methods are a manifestation of the ethical dilemma of medical staff, including the informed knowledge, respect, self-actualization, and consistency of awareness and facts [15]. In fact, conflicts also arise between mothers and medical staff in terms of the consistency of delivery methods. Researchers have found that the final delivery method was not based on mothers’ own requirements, but on the medical staff’s decisions [17]. 

As the research trend shifts from focusing on “biomedicine” (e.g., medical analgesia intervention, midwifery services, and nursing quality) [4,18] to bio-psychological-social medicine [19], the focus on women’s needs during delivery has changed from mother and child safety to subjective and humanized delivery. The influential factors of maternal satisfaction from social-psychological perception could be divided into control factors and support factors. Control factors include physical self-assessment, personal control, self-efficacy, and emotional management [1,20], and support factors include the care and support perception provided by medical staff, as well as the quality of communication between medical staff and pregnant women [12,21]. However, there has been no systematic study on the impact of social-psychological factors on maternal satisfaction in China. Chinese mothers have a low level of maternal satisfaction in Asia [22], and we propose that such low satisfaction stems from poor attitudes and support of medical staff and suppressed maternal self-efficacy. This is because a pregnant woman needs adequate knowledge, motivation, and skills to access, understand, appraise, and apply health information to make decisions about the health of herself and her unborn baby [23].

Previous research has found that maternal satisfaction could reflect the trust relationship between pregnant women and medical staff [4,11,24]. Especially in China, the delivery satisfaction of most mothers is closely related to medical staff. Women have high expectations for medical staff to support them. They prefer medical staff who are friendly, caring, and respectful, who share information, and who treat each mother equally. However, Chinese mothers rarely feel or receive support from medical staff, and they are instead sometimes yelled at, ridiculed, abused, and treated impatiently by doctors. The gap between expectations and reality frustrates maternal expectations, making women uncomfortable and reducing maternal satisfaction [25]. Moreover, the poor attitude of medical staff and little childbirth experience has made Chinese women not dare to express self-needs and forced them to rely too much on medical staff, inhibiting the positive effect of self-efficacy during the childbirth process. 

Therefore, this research tried to analyze and respond to the ethical issues surrounding the childbirth experience, especially the situation and relationship between medical staff and mothers regarding information acquisition, information awareness, plan modification, etc. Based on this research question, this article studies the influencing factors and internal mechanisms of maternal satisfaction from the perspective of social psychology and specifically discusses the relationship between the consistency of delivery mode between expectation and practice, the perceived support from medical staff and maternal self-efficacy, and the effects on maternal satisfaction. 

## 2. Literature Review and Hypotheses

### 2.1. Consistency of Delivery Mode and Maternal Satisfaction

The consistency of the delivery mode refers to the consistency between the expected delivery method and the actual delivery method. In the practice of maternal delivery, although most mothers desire their expected delivery, sometimes, the actual delivery method runs counter to their expectations [26]. Mothers’ expectations for delivery methods represents their personal needs, and when they are met, they receive high childbirth satisfaction [1]. Therefore, women presented with a consistency of delivery methods will positively evaluate the delivery process and experience [11]. However, the inconsistency of expectations versus actual delivery methods may increase psychological trauma [27] and reduce delivery satisfaction [20].

**H1.**  *The consistency of maternal delivery methods has a positive effect on women’s maternal satisfaction.*

### 2.2. Mediation Effect of Perception of Support from Medical Staff

Medical staff are expected to respect women’s feelings, provide support, and promote trust, giving more practical information about delivery, including risks of complications [28]. The respect and support of medical staff are part of the delivery process, and studies have shown that the support of medical staff has a significant impact on delivery satisfaction [11]. In hospital delivery, the support and care of medical staff meeting women’s needs should be given priority [29]. A survey by Sjogren showed that women’s biggest fear with regard to childbirth is not receiving the support of medical staff and a lack of trust in the obstetric team [30]. 

Different delivery methods will have a significant impact on the maternal perception of medical staff support. Compared with women who practice standard delivery, a cesarean section will have an adverse effect on women’s psychology, resulting in a decrease in women’s perception of medical staff support [7]. When the practice of childbirth is inconsistent with expectations, the trust in medical staff may be reduced, and the perception of their support may be lower [31]. Therefore, the inconsistency of the expectation versus the practice of the delivery mode will reduce the maternal perception of support from medical staff. 

Maternal perception of medical staff support affects maternal delivery satisfaction. In the modern obstetrics model, compared with the parturient role of patients, professional medical staff clearly occupy the prominent position of dominance and control in the delivery process [32]. During childbirth, the attitudes and behaviors of medical staff toward the parturient go beyond the effects of women’s perceptions of physical pain and medical intervention on the evaluation of childbirth [4]. The respect and support of medical staff would guarantee the women’s right to know, to participate in, and to choose freely [31]. Their support, provided continuously before and during childbirth, could promote women’s participation and expectations, thus improving women’s childbirth satisfaction [11]. However, if ignored, unsupported, abandoned, and pressured, women will have a negative perception of medical staff [33].

**H2.**  *Women’s perception of support from medical staff mediates the relationship between the consistency of delivery mode and maternal satisfaction.*

### 2.3. Moderated Mediation Effect of Women’s Self-Efficacy

Self-efficacy for childbirth has emerged as a crucial psychological construct in childbearing care. Self-efficacy is defined as a dynamic cognitive process in which women evaluate their capabilities to cope with labor and childbirth [34], and it reflects how long a woman will spend actively controlling her childbirth process [35]. Self-efficacy has been shown to be an essential social-psychological indicator of maternal satisfaction [36]. Strong self-efficacy could reduce a woman’s perception of pain, allow her to manage her own delivery process, improve the satisfaction of the childbirth experience [1], and promote the enthusiasm and initiative of parenting [37]. 

Self-efficacy is closely related to personal expectations and support perception. It has been proved that self-efficacy works as a critical element in the choice of childbirth method and the control of fear in natural childbirth [38]. Self-efficacy could increase the ability of the mother to adapt to normal childbirth and decrease the tendency for a cesarean section [39]. In China, pregnant women have low childbirth self-efficacy, and they are more likely to choose a cesarean section [17], which may be opposite to their expectations. Some studies regard childbirth as a stressful process [40], and the perception of high stress has a significant effect on low self-efficacy [22]. However, a high level of self-efficacy helps women to fight against childbirth stress. Strong self-efficacy helps to enhance maternal motivation and coping ability, to positively interact with medical staff, to improve the perception of medical staff support, and to complete non-drug painful interventions and expected delivery outcomes, thereby improving delivery satisfaction [35,41]. Based on this, this article proposes H3:

**H3.**  *Maternal self-efficacy perception has a moderated mediation effect on the relationship between the consistency of delivery mode and maternal satisfaction through perception support from medical staff. Compared with women with low self-efficacy, the positive effect of “consistency of delivery mode → perception support from medical staff → maternal satisfaction” is stronger for women with high self-efficacy.*

**Figure 1:**
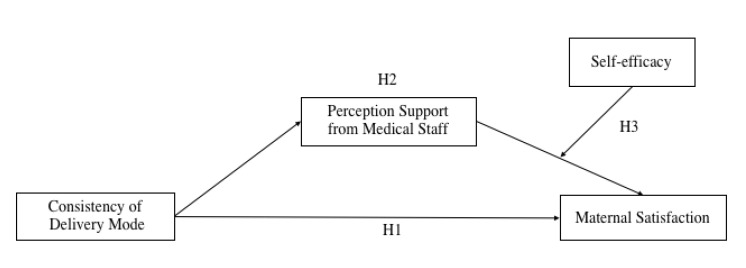
Research framework.

Based on the above hypotheses, this article explores the effect of delivery mode consistency on maternal satisfaction and the role of perception support from medical staff and maternal self-efficacy through two studies. Study 1 was a survey conducted in both urban and rural hospitals, which analyzes the main effect of the consistency of delivery mode on maternal satisfaction and the mediating effect of perception support from medical staff. Study 2 was a survey conducted online to test the relationship between the main effect of the consistency of delivery mode, the mediating effect of medical staff support, and the moderated mediation role of maternal self-efficacy. The research framework is shown in Figure 1.

## 3. Study 1

### 3.1. Materials and Methods

This study conducted a questionnaire survey for mothers who gave birth between 1 and 10 days earlier in six obstetrics and gynecology hospitals in Shanxi Province (three urban general hospitals and three rural general hospitals). In order to protect the privacy of the obstetric hospitals and mothers, the hospitals in this survey were named “A~G”. The number of births per year of the six hospitals is above 6000, which represents the comprehensive strength of the obstetrics departments of the hospitals and the actual birth situation of obstetrics departments in China.

A total of 220 questionnaires were distributed in this survey: 145 were recovered, 117 were validly answered, and the valid response rate was 69.0%. Among the 117 participants (Mean_age_ = 25.55, SD_age_ = 3.20, Max_age_ = 36, Min_age_ = 18), 81 (69.2%) gave birth for the first time, 35 (29.9%) for the second time, and 1 (0.9%) for the third time. Regarding geographical distribution, there were 48 urban women (41%) and 69 rural women (59%) (refer to Table 1 for more information on demographics).

At the beginning of the survey, all participants signed an informed consent form. They were guaranteed anonymity and allowed to discontinue the survey at any time. The participants were informed that the survey consisted of multiple sections to understand their satisfaction with delivery. After the survey, each participant could receive a bag of laundry as a reward. 

The questionnaire included four parts: (1) Delivery mode. The participants were asked to answer the following questions about the delivery mode: the actual delivery mode (1 = vaginal delivery without anesthesia, 2 = painless delivery, 3 = caesarean section, 4 = suction delivery, 5 = forceps delivery). During data analysis, we categorized the delivery mode into 0 = caesarean section, 1 = vaginal delivery (including the options of 1, 2, 4, and 5, all childbirth through the women’s birth canal and vagina, with and without medical intervention). They also answered the consistency of delivery method (1 = yes; 0 = no). (2) Perception of support from medical staff was adapted from Matsuoka et al. (2009), with four items to evaluate the attitude and behavior of medical staff (a = 0.908). The items consisted of “The medical staff provide me with continuous care and assistance”, “The obstetricians provide me with abundant delivery knowledge and guidance during pregnancy”, “During childbirth, the medical staff especially respected and valued me,” and “After giving birth, the medical staff provided me with physical and mental care and shared parenting knowledge”, using a 5-point Likert scale (1 = very dissatisfied, 5 = very satisfied). These items were proved to combine as one factor (KMO = 0.849, *p* < 0.000) and explain 78.702% of variance. (3) Maternal satisfaction with the delivery experience was measured using a 5-point Likert scale (1 = very dissatisfied, 5 = very satisfied). (4) Demographics were collected, including age at first birth, age at second childbirth, occupation, education level, and living area (1 = urban, 0 = rural).

### 3.2. Results

Common method bias check. Given the nature of a single-shot cross-sectional survey, we first checked whether there was a common method bias before the formal data analysis. Harman’s one-factor analysis was conducted [42] by including all of the items of key variables for an exploratory factor analysis using a maximum likelihood solution. In Study 1, we identified three factors and found the first factor accounted for 33.558%, which is below the criterion of 50% [43]. Therefore, there is no common method bias in Study 1. 

Description of delivery mode and consistency. Among the 117 participants, 78 (66.7%) had vaginal deliveries (including 74 with vaginal delivery, 3 with painless delivery, and 1 with forceps delivery), and 39 (33.3%) had cesarean sections. As for the delivery mode consistency, 81 women claimed that they experienced their desired delivery method (69.2%), and 36 (30.8%) women had a delivery method inconsistent with their expected method. Among the 36 mothers, 20 (17.1%) conducted the inconsistent delivery method because of abnormal conditions during childbirth; the others were forced to choose a cesarean section because of the doctors’ advice (11, 9.4%), the woman’s changed willingness (4, 3.4%), or their family’s suggestion (1, 0.9%). 

Main effect. The mean, standard deviation, and correlation analysis of key variables in Study 1 are shown in Table 2. Regression analysis was conducted with the consistency of delivery mode as the independent variable and maternal satisfaction as the dependent variable. The results showed that the consistency of delivery mode could positively increase maternal satisfaction (β = 0.413, t = 4.863, *p* < 0.000), and it still had a significantly positive effect (β = 0.419, t = 4.642, *p* < 0.000) on maternal satisfaction after controlling for some demographics, numbers of delivery, and numbers of medical intervention (results referred to Table 3). In addition, compared with women who expected and practiced a consistent delivery mode (M = 4.05, SD = 0.92), women who expected and practiced an inconsistent delivery mode had a lower satisfaction with delivery (M = 3.06, SD = 1.22). Thus, H1 was supported.

Mediation. We predicted that the perception of support from medical staff would mediate the effect of the consistency of delivery mode on maternal satisfaction. A 5000 resampling bootstrapping mediation analysis using consistency of delivery mode as the predictor, perception of support from medical staff as the mediator, and maternal satisfaction as the dependent variable (Hayes 2018, Model 4) confirmed this prediction. The analysis revealed a significant omnibus index of mediation (Effect = 0.2638 SE = 0.1257, 95% CI: (0.0303, 0.5310)). Thus, H2 was supported.

### 3.3. Discussion

Based on the participants’ self-reported survey results in six hospitals, Study 1 proved that the consistency of delivery mode between expectation and practice behavior had a positive main effect on maternal satisfaction, and a consistency of delivery mode showed a higher satisfaction than that with an inconsistent delivery mode. The mechanism of the above effect was mediated by women’s perception of support from medical staff. Therefore, both H1 and H2 were supported. However, there are two shortcomings in Study 1. On the one hand, the support of medical staff could be further classified, such as the material, emotional, informational, and behavioral support provided by medical staff. On the other hand, women might have the power to control the efficacy of their bodies and influence the whole process of delivery and maternal satisfaction, which may work as a moderator. Study 2 would further address the above issues.

## 4. Study 2

In Study 2, two more issues were investigated. One was the updated measurement of the perception of support from medical staff, namely, the Breastfeeding Self-Efficacy Scale Short Form (BSES-SF). The other was the inclusion of women’s self-efficacy to test whether it played a moderating role in the relationship between the consistency of delivery mode, perception of support from medical staff, and maternal satisfaction.

### 4.1. Participants

A total of 237 female participants who had children were recruited from the sample database on the Credamo platform (https://www.credamo.com/#/ accessed on July and August 2022); these women had not joined Study 1. Among the 237 responses, 32 qualified responses were rejected through automatic screening and attention detection, and finally, 205 valid responses were recovered. All the participants had their first delivery at the average age of 26.09 (SD = 2.53, N = 205), and 57 of them had their second delivery experience at the average age of 30.02 (SD = 2.32, N = 57). Table 4 shows the basic demographics of participants, including geographical distribution, education, occupation, and monthly household income.

### 4.2. Procedures and Measures

Study 2 was a survey to test the relationship between the consistency of delivery mode, perception of support from medical staff, mothers’ self-efficacy during childbirth, and maternal satisfaction. Mature scales verified with high reliability and validity were chosen and subsequently translated from English to Chinese, following the back-translation process [44]. 

Participants were invited to read the informed consent online at the beginning of the survey. Then, they were asked to answer five parts of a questionnaire. After their answers were checked and qualified, they would receive 5 yuan in RMB as a reward. 

The five parts of the questionnaire included: (1) Delivery mode. The participants were asked to answer two questions, namely, the “delivery method in the last delivery experience” and “expected delivery method in the latest delivery experience”, with four options: 1 = vaginal delivery without medical intervention, 2 = vaginal delivery with medical intervention (such as oxytocin, artificial water breaking, lateral incision, etc.), 3 = painless delivery (epidural anesthesia), 4 = cesarean section. Just like Study 1, we combined the choice of 1 and 3 as a new variable, vaginal delivery, compared with the other kind of delivery mode, the caesarean section. (2) Perception of support from medical staff, adapted from the Brinell Childbirth Support Perception Scale (The Breastfeeding Self-Efficacy Scale Short Form) [45]. The scale has 25 items, such as “the medical staff helped me to familiarize myself with the environment, such as giving me a detailed introduction to the delivery environment”, “the medical staff cared about me, being very kind and friendly, and let me feel honored”, “The medical staff communicated my needs and wishes to the doctor and other hospital staff”, “The medical staff encouraged my husband/main attendant to participate in the production process and gave him a positive response”. A 5-point Likert scale was used (1 = very dissatisfied, 5 = very satisfied) with good reliability (α = 0.962). (3) Maternal perceptions of self-efficacy were assessed using the Childbirth Self-Efficacy Inventory (CBSEI) [40], with good reliability (α = 0.936). Participants were asked to answer 16 questions, such as “I can relax my body in some ways”, “I can maintain self-control during childbirth”, and “I can give myself positive guidance”, with a 5-point Likert scale (1 = not at all, 2 = rarely, 3 = sometimes, 4 = often, 5 = absolutely). (4) Maternal satisfaction, using a 5-point Likert scale (1 = very dissatisfied, 5 = very satisfied). (5) Demographic variables, including gender, age, occupation, highest educational background, living area (urban/rural), and monthly household income.

### 4.3. Results 

Common method bias check. Just like in Study 1, a common method bias was conducted in Study 2. The results showed that 12 factors emerged with eigenvalues larger than 1.00, indicating that more than one factor underlay the data. In addition, the first factor accounted for only 31.298% of the total variance, suggesting that the common method variance may not be a severe concern in the present study.

Description of delivery mode and consistency. In terms of the expectant delivery method, 77 (37.6%) women expected a vaginal delivery without medical intervention, 39 (19.0%) expected a vaginal delivery with medical intervention, 67 (32.7%) expected a painless delivery, and 22 (10.7%) expected a cesarean section. However, as for the actual delivery method, 33 (16.1%) had a vaginal delivery without medical intervention, 96 (46.8%) had a vaginal delivery with medical intervention, 52 (25.4%) delivered by caesarean section, and 24 (11.7%) had a painless delivery. In terms of the consistency of delivery methods, 92 (44.9%) had consistent delivery methods between expectations and practical experience; however, 113 (55.1%) had an inconsistent experience. Among the 92 cases with consistency of delivery mode, 22 (23.9%) experienced vaginal delivery without medical intervention, 30 (32.6%) had medical intervention, 20 (21.7%) had a painless delivery, and 20 (21.7%) delivered by cesarean section. It can be seen that most mothers expected natural labor without medical intervention and painless delivery, but they failed to choose and practice the corresponding delivery method as scheduled.

Correlation. Factor analysis was conducted for self-efficacy and the perception of support from medical staff. Self-efficacy was proved to combine as one factor (KMO = 0.946, *p* < 0.000), and the perception of support from medical staff had three sub-factors (KMO = 0.955, *p* < 0.000), which were named as the perception of emotional support, behavioral support, and support given to partners. The mean, SD, and correlation of key variables are shown in Table 5. Demographic variables (education, monthly household income, age at first birth) were not significantly associated with delivery satisfaction and were therefore not analyzed as control variables.

Main effect. Using SPSS 26.0, regression analysis was performed, with consistency of delivery mode (0 = no, 1 = yes) as the predictor and maternal satisfaction as the outcome variable. Meanwhile, the influence of occupation, education, area (1 = city, 2= rural), monthly household income, number of deliveries, and the age at delivery were controlled in Model 2. The results indicated that the consistency of delivery mode could increase women’s maternal satisfaction (β = 0.323, t = 2.623, *p* = 0.011) in Model 1 and was still robust in Model 2 (β = 0.342, t = 2.631, *p* = 0.011). Results are shown in Table 6.

Mediation effect. A 5000-resampling bootstrapping mediation analysis using consistency of delivery mode as X, perception of support from medical staff as M, and maternal satisfaction as Y (Model 4) [46] was conducted to test H2. The analysis revealed a significant omnibus index of mediation (effect = 0.2773, SE = 0.0716, 95% CI = (0.1465, 0.4303)).

Moderated Mediation effect. Following Model 14 of the PROCESS Macro [46], we performed a 5000-resampling bootstrapping-moderated mediation analysis, with consistency of delivery mode as the independent variable, perception support from the medical staff as the mediator, women’s self-efficacy as the moderator, and maternal satisfaction as the dependent variable. The results revealed a significant index of moderated mediation (effect = 0.1123, SE = 0.0482, 95% CI = (0.0331, 0.2252)). A simple slope test showed that for women with low levels of self-efficacy (M − 1 SD = 2.9141), the mediating effect was significant for the path “consistency of delivery mode → perception of support from medical staff → maternal satisfaction” (effect = 0.2030, SE = 0.0655, 95% CI = [0.0968, 0.3591)), and for women with high levels of self-efficacy (M + 1 SD = 4.1536), the positive effect was strengthened significantly (effect = 0.3422, SE = 0.0906, 95% CI = (0.1822, 0.5430)).

### 4.4. Discussion

Higher delivery satisfaction depends not only on the consistency of expected delivery mode and practice, but also on the strength of the perception of medical staff’s support and the mother’s self-efficacy. Although the substantive care and guidance of attendants by medical staff are important during childbirth, mothers perceiving to be respected, informed, and to actively participate decision making is essential to build a positive childbearing experience. As Harris and Ayers have pointed out, being neglected, unsupported, under mental stress, or not having attention paid to the mental demands of mothers will lead to negative experience evaluations [33]. Therefore, women with a high level of self-efficacy would perceive more support from medical staff, and this increases maternal satisfaction. Thus, H1, H2, and H3 were approved.

## 5. Conclusions and Discussion

### 5.1. Conclusions

Two studies confirmed our hypothesis that women with a consistent delivery mode would be more satisfied with their childbirth experience (H1); women’s perception of support from medical staff mediated the relationship between the consistency of delivery mode and maternal satisfaction (H2); and maternal self-efficacy was a moderator for the above mediation effect; namely, for the women with high levels of self-efficacy, the positive effect of the path “consistency of delivery mode → perception support from medical staff → maternal satisfaction” was stronger (H3). 

Existing research about the effect of delivery mode on delivery satisfaction has focused on practical behaviors and experiences, such as the cesarean section, vaginal delivery, and medical intervention [15,16]. This study showed that the delivery satisfaction of Chinese women depends not only on the delivery method they experienced, but also on the consistency between their expectations and experience. 

The positive correlation between the consistency of delivery mode between expectations and experience and maternal satisfaction is mediated by the perception of support from medical staff. Medical staff’s support could be categorized into three aspects: the perception of emotional support, behavioral support, and support given to partners, which confirms that during childbirth, women need guidance and companionship, rather than medical treatments that alter, suppress, or accelerate bodily processes [26]. However, in the modern obstetric mode, quantitative indicators and standardized operations have turned the colorful delivery experience into an assembly line that can be completed with a few scissors or a single operation [47]. During the delivery process, mothers need to be respected, rather than isolated, disturbed, judged, blamed, reprimanded, denied, urged, despised, and watched. The perception of support from medical staff will help the mother to make her own choice, which will eventually make her feel more confident and satisfied with the delivery experience. 

Childbirth is not only a biological process for women, but also an essential part of their well-being. Therefore, in addition to the perception of external support from the medical staff, the active factors of self-control and regulation, namely, self-efficacy, are also indispensable. The results of this research revealed that self-efficacy plays a moderated mediation role. Compared with mothers with low self-efficacy, the relationship between the consistency of delivery mode expectation and actual experience, perception support from medical staff, and maternal satisfaction was stronger for mothers with high self-efficacy. A survey showed that medical staff’s inadequate support and excessive control only accounted for 12% of the unsatisfaction with delivery, and pregnant women’s poor self-efficacy was the most important factor affecting the evaluation of childbirth [28]. Presently, the status of the self-efficacy of pregnant women in China is not ideal. On the one hand, prenatal education mainly focuses on the anxiety guidance of avoiding danger and neglects mobilizing the positive subjective initiative of pregnant women. On the other hand, medicalization incorporates childbirth into the scope of biomedicine, assigns the mother to the role of patient, and strengthens the responsibility of mothers to protect their babies from disease. Both of the above conditions negatively influence the formation and function of maternal self-efficacy. It can be seen that maternal self-efficacy is very important for practicing delivery methods, as expected, enhancing communication with medical staff, increasing the perception of support from medical staff, and further improving delivery satisfaction. 

### 5.2. Theoretical Contribution

The theoretical contribution of this paper has two aspects. First, this research analyzed the relevant factors affecting maternal satisfaction from the perspective of social psychology. Extant literature has focused on the influence of objective medical conditions, equipment updates, and other elements on maternal satisfaction from the “biomedicine” perspective [19]. The results claimed that Chinese women’s evaluation of childbirth experience comes from the consistency of expected delivery mode and practical behavior, medical ‘and mothers’ self-efficacy. This research advocated improving the level and quality of medical services from the perspective of maternal mental health and psychological needs, from a perspective of “bio-psychological-social medicine” [19].

Second, this paper enriches the research issues of medical management ethics on childbirth. Doctors and medical staff, as experts on the body and ailments, have been thought to be in the essential position to decide what to do and how to do it for women during childbirth. According to bioethics, the basic principles of ethics in healthcare emphasize respect for persons, namely, the acknowledgment of individual autonomy. Therefore, women should have the right to decide what is done to their bodies [48] during the prenatal, intrapartum, and postpartum periods [49]. However, the literature about childbirth ethics in the intrapartum period only concerns the practical use of medication and obstetrical procedures, such as whether the women receive adequate information on consent [50] and delivery [51], whether they use medication to relieve pain [52], and whether they undergo a surgical birth (or cesarean delivery) [53]. In this research, however, women’s ethical autonomy during childbirth was represented by the consistency of delivery mode between women’s expectations and experience, the perception of support from medical staff, and women’s self-efficacy. The findings are consistent with a bioethics perspective and extend the research attention of childbirth ethics to woman-centered childbirth management services [54]. Women’s participation in decision making should be addressed and ensured, and the quality of care should be improved to build accountability and communication between medical providers, women, and their families.

### 5.3. Practical Implications

Although medical services are constantly improving in China, the humanistic care and service awareness of medical staff toward patients are still insufficient. According to the results of this article, maternal satisfaction could be improved in two ways. The first is to enhance the mother’s perception of support from medical staff. Therefore, medical staff should be trained on their service attitudes and practices to interpret test results, in order to promote the correct and positive transmission of relevant knowledge to mothers. According to The Network for Improving Quality of Care for Maternal, Newborn, and Child Health, promoted by the World Health Organization in 2017, medical staff should be taught to facilitate learning, share knowledge, and generate evidence on the quality of care [55]. Both the type of information and the tone of the information conveyed by medical staff play an essential role to cultivate maternal awareness of mothers’ childbirth, meet their expectations, and improve their satisfaction [56]. It is crucial for medical staff to improve the quality of care, adhering to the principles of respect, self-love, equality, and openness, and to establish a smooth information communication channel and sharing mechanism among pregnant women, families, medical staff, etc. 

The other possible improvement is to encourage maternal self-efficacy to participate actively in the delivery process. Improving maternal self-efficacy to participate in health promotion behaviors and reversing the self-assessment of physical health is an excellent way to promote benign communication between maternal and medical staff and to enhance delivery satisfaction. Specifically, medical staff would provide positive psychological hints to mothers, guide them to have a positive assessment of maternal behavior, and improve their awareness and ability to participate in their own childbirth decision-making process.

### 5.4. Limitations and Future Research Directions 

This article demonstrated the influential factors in maternal satisfaction through two studies with cross-sectional data. Study 1 was conducted by surveying women who gave birth within ten days, and Study 2 was performed by recalling their most recent childbirth experience. Future research can study maternal satisfaction at multiple time points throughout the process of pregnancy and delivery by long-term follow-up. In addition, the maternal self-efficacy of Study 2 was measured by questionnaires. In future research, between-subject behavioral experiments could be used to prime different levels of mothers’ self-efficacy, in order to explore the moderating effect of maternal self-efficacy on maternal satisfaction.

## Figures and Tables

**Table 1:** Description of participants’ demographics in Study 1.

Demographics	N	Percentage (%)
Area		
Urban	48	41.03
Rural	69	58.97
Education		
Primary school	9	7.69
Junior high school	26	22.22
High school	20	17.09
Bachelor’s degree	58	49.57
Masters’ degree and above	4	3.42
Occupation		
Public institutions	54	46.15
Private/foreign-invested enterprises	25	21.37
Unemployed	31	26.50
Others	7	5.98

Note: N = 117.

**Table 2:** Means, standard deviations, and correlation for key variables in Study 1.

Variable	M	SD	1	2
1. Consistency of delivery mode	-	-		
2. Perception of support from medical staff	4.02	0.96	0.205 *	
3. Maternal satisfaction	3.74	1.12	0.413 **	0.598 **

Note: * *p* < 0.05, ** *p* < 0.01; consistency of delivery mode: 0 = no, 1 = yes.

**Table 3:** Main effect of consistency of delivery mode on maternal satisfaction in Study 1.

Maternal Satisfaction	Model 1		Model 2	
	β	t	β	t
Consistency of delivery mode	0.413 **	4.836	0.419 **	4.642
Age of first delivery			−0.086	−0.741
Area			0.048	0.376
Education			−0.002	−0.018
Occupation			0.111	0.969
Number of deliveries			−0.108	−1.143
Number of medical interventions			0.109	1.220
R^2^	0.170		0.198	
∆R^2^	0.163		0.146	
F	23.385		3.818	

Note: * *p* < 0.05, ** *p* < 0.01; consistency of delivery mode: 0 = no, 1 = yes.

**Table 4:** Description of participants’ demographics in Study 2.

	N (%)	Percentage
Area		
Urban	66	47.1
Rural	74	52.9
Education		
Junior high school	2	1.0
High school	7	3.4
Specialist school	35	17.1
Bachelor’s degree	146	71.2
Masters’ degree	9	4.4
Doctoral degree	6	2.9
Occupation		
Student	1	0.5
State-owned enterprise	44	21.5
Private enterprise	90	43.9
Foreign companies	20	9.8
Institutions	24	11.7
Medical staff	3	1.5
Civil servant	4	2.0
Self-employed	14	6.8
Unemployed	5	2.4
Monthly household income		
<5000	10	4.9
5000–9999	29	14.1
10,000–14,999	61	29.8
15,000–19,999	44	21.5
20,000–29,999	15	7.3
≥30,000	7	3.4
Number of deliveries		
Once	148	72.2
Twice	57	27.8

Note: N = 205.

**Table 5:** Means, standard deviations, and Pearson correlation coefficients for all variables.

Variable	M	SD	1	2	3
1. Consistency of delivery mode	-	-			
2. Perception support from medical staff	4.09	0.63	0.26 **		
3. Self-efficacy	3.53	0.62	−0.76	0.51 **	
4. Maternal satisfaction	3.98	0.94	0.34 **	0.61 **	0.31 **

Note: ** *p* < 0.01; consistency of delivery mode: 0 = no, 1 = yes.

**Table 6:** Main effect of consistency of delivery mode on maternal satisfaction in Study 2.

Maternal Satisfaction	Model 1		Model 2	
	β	t	β	t
Consistency of delivery mode	0.323 *	2.623	0.342 *	2.631
Occupation			−0.166	−1.111
Education			−0.081	−0.505
Area			0.294 *	2.251
Monthly household income			0.392 **	2.897
Number of deliveries			0.040	0.281
Age of first delivery			−0.095	−0.444
Age of second delivery			0.147	0.694
R^2^	0.077		0.277	
∆R^2^	0.061		0.159	
F	4.678		2.348	

Note: * *p* < 0.05, ** *p* < 0.01.

## Data Availability

Some or all data and models that support the findings of this study are available from the corresponding author upon reasonable request.
